# Genetic diversity of the enterohaemolysin gene (*ehxA*) in non-O157 Shiga toxin-producing *Escherichia coli* strains in China

**DOI:** 10.1038/s41598-018-22699-7

**Published:** 2018-03-09

**Authors:** Shanshan Fu, Xiangning Bai, Ruyue Fan, Hui Sun, Yanmei Xu, Yanwen Xiong

**Affiliations:** 1 State Key Laboratory of Infectious Disease Prevention and Control, National Institute for Communicable Disease Control and Prevention, Chinese Center for Disease Control and Prevention, Changping, Beijing, China; 2 Collaborative Innovation Center for Diagnosis and Treatment of Infectious Diseases, Hangzhou, Zhejiang Province China

## Abstract

Non-O157 Shiga toxin-producing *Escherichia coli* (STEC) is increasingly recognized as an important enteric foodborne pathogen. The hallmark of the disease is the production of Shiga toxins; however, there are other virulence factors that contribute to the pathogenesis of STEC. This study aimed to investigate the prevalence and genetic diversity of the enterohaemolysin gene, *ehxA*, among non-O157 STEC strains from human, animal, and food sources. The *ehxA* gene was amplified from 138 (31.8%) of 434 non-O157 STEC strains, among which 36 unique *ehxA* sequences were identified. Based on *ehxA* sequence analysis, three phylogenetic *ehxA* groups (I II, and III) were determined. Correlations between *ehxA* groups and sources, serotypes, and virulent gene profiles were observed. The *ehxA* group II strains were mostly diarrhoeal patient-derived and may demonstrate higher pathogenic potential compared with the *ehxA* group I and group III strains. Five types of replicons (I1-Ig, FIB, K, F, and B/O) were identified in the 138 *ehxA*-positive strains, and 3.6%, 5.8%, and 52.2% of the strains harboured *toxB*, *katP* and *espP* genes, respectively, implying marked genetic diversity of *ehxA* containing plasmids in non-O157 STEC strains. Sequence-based *ehxA* genotyping might be important in modern strain typing and in epidemiological surveillance of non-O157 STEC infections.

## Introduction

Shiga toxin-producing *Escherichia coli* (STEC) is an important enteric foodborne pathogen causing mild human diarrhoea, haemorrhagic colitis (HC), and fatal haemolytic uremic syndrome (HUS) worldwide^1^. More than 400 serotypes have been detected in STEC, and O157:H7 is regarded as the most predominant and virulent serotype associated with severe human illness^2^. Nevertheless, recent studies revealed that non-O157 STEC serotypes, with O26, O45, O103, O111, O121, and O145 being the top six serogroups, are responsible for increasing numbers of outbreaks or sporadic cases worldwide^3^. Domestic or wild animals are the main natural reservoirs of STECs. Humans are the accidental host of STEC through contact with animals or ingestion of contaminated meat, milk, vegetables, fruit, and water^4,5^.

Shiga toxin (Stx) is regarded as the most critical virulence factor, which can be divided into two types: Stx1 and Stx2^6^. Several subtypes and variants for each type have been described^7^. Another virulence factor, intimin (*eae*), is located on the locus of enterocyte effacement (LEE), and induces characteristic histopathological lesions, referred to as attaching and effacing lesions^8^. In addition, haemolysin plays an important role in STEC pathogenicity. To date, four different types of haemolysin (*hlyA*, *ehxA*, *sheA*, and *e-hlyA*) have been identified in *E. coli*, among which the plasmid-carried enterohaemolysin (*ehxA*) is widespread in STEC strains and is frequently associated with diarrhoeal disease and HUS^9–11^. The presence of enterohaemolysin correlates with that of Shiga toxin; therefore, it has been suggested as an epidemiological marker for the rapid and simple detection of STEC strains^12^. Six genetically distinct *ehxA* subtypes (A to F) were described in *E. coli* using PCR and restriction fragment length polymorphism (RFLP) analysis^10^.

The complete *ehxA* gene is about 3000 base pairs and resides in the *ehx* cluster, which contains four genes in the order of *ehxC*, *ehxA*, *ehxB*, and *ehxD*^13^. *ehxC* is associated with haemolysin activation, and *ehxB* and *ehxD* are related to the secretion of haemolysin^13,14^. In O157 STECs, the *ehx* cluster is located on a plasmid called pO157, a non-conjugative F-like plasmid with ranging in size from 92 to 104 kb. pO157 is a dynamic structure that contains other putative virulence-related factors, such as a catalase-peroxidase (*katP*) that increases the ability to colonize the host intestine in the absence of oxygen; a serine protease (*espP*) that influences the colonization and adhesion to intestinal epithelial cells; and a putative adhesin (*toxB*) that enhances adhesion by increasing the secretion of type three secretion system (TTSS)^15–17^. These genes may have important functions in the pathogenicity of STECs; however, their roles are not fully understood^18^.

In our previous studies, we systematically investigated the prevalence of STEC from various sources and geographical areas in China, and collected a broad variety of non-O157 STEC strains from cattle, goats, pigs, yaks, marmots, pika, antelopes, food, diarrhoeal patients, and healthy carriers^19^. The objective of the present study was to investigate the prevalence and genetic diversity of *ehxA* genes in correlation with the sources, serotypes, virulence profiles, haemolytic activities, and potential pathogenicity among various non-O157 STEC strains. Furthermore, using sequence-based analysis instead of the traditional PCR-RFLP method, which might produce possible misinterpretation, we intended to genotype the *ehxA* genes with high stability and accuracy, thus gaining a better understanding of their genetic diversity and relatedness.

## Results

### Proportion of *ehxA* in the non-O157 STEC collection

A total of 434 non-O157 STEC strains were screened for the presence of the *ehxA* gene. *ehxA* tested positive in 138 (31.8%) strains isolated from different sources including humans, animals, and foods: diarrhoeal patients (9), healthy carrier (1), yaks (66), pika (15), antelopes (4), marmots (4), goats (12), beef cattle/cow (6), pig (1), raw mutton (10), raw beef (8), raw chicken meat (1), and raw duck meat (1) (Tables 1 and S1).

**Table 1:** The origin and location of 434 non-O157 STEC isolates used in this study.

Source	Location	Sampling year	No. of samples	No. of *stx-*positive	No of *ehxA*-positive (%)*
Goat	Henan	2009	N/A	28	12 (42.9%)
Yak	Qinghai	2012	728	128	66 (51.6%)
Plateau Pika	Qinghai	2012, 2012, 2015	1116	22	15 (68.2%)
Marmots	Qinghai	2012, 2013	335	8	4 (50.0%)
Tibetan antelope	Qinghai	2014	505	5	4 (80.0%)
Pig	Chongqing, Beijing, Guizhou, Shandong, Heilongjiang	2011, 2012, 2013, 2015	1181	135	1 (0.7%)
Cattle/Cow	Shandong, Heilongjiang	2009, 2013, 2015	605	14	6 (42.9%)
Raw meat	Beijing, Sichuan	2013, 2014	853	60	20 (33.3%)
Diarrhoeal patient	Henan, Shenzhen, Shanghai, Sichuan	2010, 2012, 2013, 2014, 2016	N/A	30	9 (33.3%)
Healthy carrier	Qinghai, Shenzhen	2013, 2014	2058	4	1 (25.0%)
Total				434	138 (31.8%)

N/A: The number of samples was not applicable. *The percentage of *ehxA* gene was detected in the *stx*-positive strains.

### Diversity of *ehxA* and *ehxCABD*

A total of 138 complete *ehxA* sequences were obtained and the sizes were all 2,997-bp. Among the 138 *ehxA* sequences, 36 unique *ehxA* sequences were identified, which were nominated as *ehxA* genotype 1 to genotype 36 (Table S2). The nucleotide identities among the 36 *ehxA* genotypes ranged from 96 to 99%.

Forty-two *ehxCABD* cluster sequences were obtained from the whole genome sequences. Similar to *ehxA*, the 42 complete *ehxCABD* clusters showed high similarity. The identities ranged from 93 to 100% for *ehxC*, 97 to 100% for *ehxB*, and 96 to 100% for *ehxD*.

### *ehxA* groups based on the phylogenetic trees

**Figure 1:**
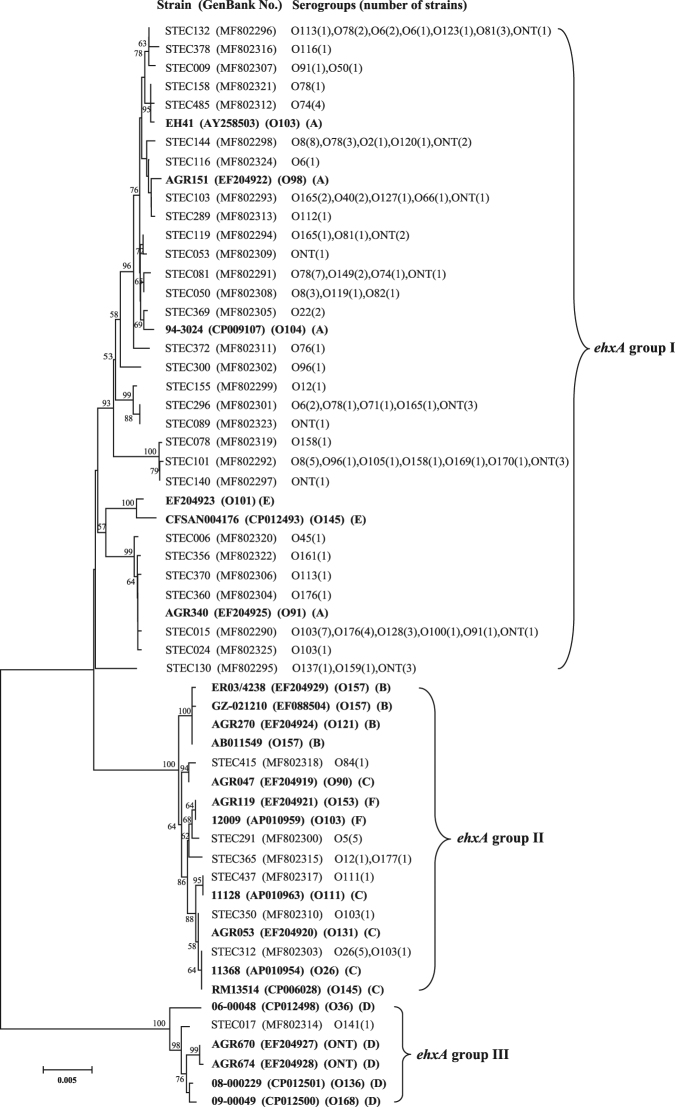
Phylogenetic relationships of *ehxA* sequences based on the neighbour-joining method. Thirty-six unique *ehxA* sequences were obtained in this study. The serogroups for each *ehxA* genotype (representative strain) are given. Twenty-two sequences of six *ehxA* PCR-RFLP (polymerase chain reaction-restriction fragment length polymorphism) subtypes A to F downloaded from GenBank are indicated in bold. Bootstrap values > 50% are shown at the branch points.

A neighbour-joining tree was constructed using the 36 unique *ehxA* sequences obtained in this study and 22 reference *ehxA* sequences (Fig. 1). The phylogenetic trees reconstructed using the neighbour-joining algorithm and the maximum-likelihood algorithms were quite similar (Fig. S1), which demonstrated that all *ehxA* sequences fell into three phylogenetic groups, named group I, group II, and group III. The sequence similarities within each group were higher than 98%. The majority (87.7%, 121 out of 138) of our *ehxA*-positive STEC strains clustered into group I; 16 (11.6%) strains were placed into group II; and only one strain belonged to group III (Fig. 1).

### STEC origins correlated with the *ehxA* groups

In this study, most animal-derived strains belong to group I, with exception of two marmot strains and one cattle strain that belonged to group II, and one goat strain that belonged to group III. Notably, all nine diarrhoeal patient-derived strains belonged to group II and one healthy carrier strain belonged to group I. Food-derived strains belonged to group I or II. Certain *ehxA* groups were associated with non-O157 STEC origins (*P* < 0.05) (Table 2).

**Table 2:** The origins and virulence factors associated with *ehxA* groups in 138 non-O157 STEC strains.

*ehxA* groups	No. of strains	No. of strains with virulence factor (s) from different sources					
		Animal		Human		Food	
		*stx*	*stx* + *eae*	*stx*	*stx* + *eae*	*stx*	*stx* + *eae*
**I**	121	102	2	1	0	16	0
**II**	16	2	1	1	8	0	4
**III**	1	1	0	0	0	0	0
Total	138	105	3	2	8	16	4

### STEC serotypes in different *ehxA* groups

In total, 118 *ehxA*-positive non-O157 STEC strains were typed into 43 different O serogroups and 20 strains were O-untypable (ONT). *ehxA* groups I, II, and III contained 37, seven and one serogroup(s), respectively. Only strains of serogroup O103 and O12 were observed in both group I and II (Table 3); however, they could be further distinguished by their H types. Strains of O103:H8 and O12:H8 were in *ehxA* group I, while strains O103:H25 and O12:H-untypable were in group II (Table S2).

**Table 3:** Serogroups, virulence factors, origin, and plasmid Inc (incompatibility) associated with *ehxA* groups.

Serogroup	Number	*stx*			*eae*	Origin			Inc (number)	*ehxA* group		
		*stx*_1_	*stx*_2_	*stx*_1_ + *stx*_2_		H	A	F		I	II	III
O2	1	0	1	0	0	0	1	0	FIB(1);F(1)	1	0	0
O5	5	5	0	0	3	2	2	1	FIB(5);F(3)	0	5	0
O6	5	3	2	0	0	0	5	0	FIB(5);F(4);K(1)	5	0	0
O8	16	3	5	8	0	0	16	0	I1-Ig(1);FIB(15);F(16);B/O(1)	16	0	0
**O12**	**2**	**1**	**1**	**0**	**1**	**0**	**1**	**1**	**FIB(1);F(2);B/O(1)**	**1**	**1**	**0**
O22	2	0	1	1	0	0	1	1	FIB(2);F(2)	2	0	0
O26	5	5	0	0	5	5	0	0	FIB(5); B/O(2)	0	5	0
O40	2	0	2	0	0	0	0	2	FIB(2);F(2);B/O(1)	2	0	0
O45	1	0	0	1	0	0	1	0	FIB(1);K(1)	1	0	0
O50	1	1	0	0	0	0	1	0	FIB(1);F(1)	1	0	0
O66	3	1	2	0	0	0	3	0	FIB(2);F(3);K(2)	3	0	0
O71	1	0	1	0	0	0	1	0	FIB(1);F(1)	1	0	0
O74	5	0	4	1	0	0	5	0	FIB(3);F(4);K(4)	5	0	0
O76	1	1	0	0	0	0	0	1	K(1)	1	0	0
O78	13	0	11	2	2	0	13	0	FIB(11);F(5);K(3)	13	0	0
O81	4	0	4	0	0	0	4	0	FIB(4);F(3);K(1)	4	0	0
O82	1	1	0	0	0	0	1	0	FIB(1);F(1);B/O(1)	1	0	0
O84	1	1	0	0	1	1	0	0	FIB(1);F(1)	0	1	0
O91	3	0	0	3	0	1	2	0	FIB(3);F(3)	3	0	0
O96	2	0	2	0	0	0	1	1	FIB(1);F(1)	2	0	0
O100	1	0	1	0	0	0	1	0	FIB(1);F(1);K(1)	1	0	0
**O103**	**10**	**10**	**0**	**0**	**2**	**0**	**8**	**2**	**FIB(10);F(8);K(3); B/O(5)**	**8**	**2**	**0**
O105	1	0	1	0	0	0	1	0	FIB(1);F(1);K(1)	1	0	0
O111	1	1	0	0	0	1	0	0	FIB(1);F(1);B/O(1)	0	1	0
O112	1	1	0	0	0	0	1	0	FIB(1);F(1)	1	0	0
O113	2	1	0	1	0	0	1	1	FIB(2);F(2)	2	0	0
O116	1	0	0	1	0	0	0	1	FIB(1);K(1)	1	0	0
O119	1	1	0	0	0	0	1	0	F(1);B/O(1)	1	0	0
O120	1	0	0	1	0	0	1	0	FIB(1);F(1)	1	0	0
O123	1	0	0	1	0	0	1	0	FIB(1);F(1)	1	0	0
O127	1	0	0	1	0	0	1	0	FIB(1);F(1)	1	0	0
O128	3	0	0	3	0	0	0	3	FIB(3);F(2);K(1); B/O(2)	3	0	0
O137	1	0	0	1	0	0	1	0	F(1);B/O(1)	1	0	0
O141	1	0	1	0	0	0	1	0	FIB(1);F(1)	0	0	1
O149	2	0	2	0	0	0	2	0	FIB(2);F(1)	2	0	0
O158	2	0	2	0	0	0	2	0	FIB(2);F(2)	2	0	0
O159	1	0	0	1	0	0	1	0	Negative	1	0	0
O161	1	1	0	0	0	0	0	1	Negative	1	0	0
O165	4	2	1	1	0	0	4	0	FIB(3);F(3);K(1)	4	0	0
O169	1	0	1	0	0	0	1	0	FIB(1);F(1)	1	0	0
O170	1	0	1	0	0	0	1	0	FIB(1);F(1)	1	0	0
O176	5	4	0	1	0	0	0	5	FIB(4);F(4);K(1)	5	0	0
O177	1	0	1	0	1	0	1	0	FIB(1);F(1)	0	1	0
ONT	20	8	6	6	0	0	20	0	FIB(20);F(19);K(2); B/O(3)	20	0	0

H: human; A: animal; F: food. Strains of serogroup O103 and O12 in both group I and group II are indicated in bold.

### Co-occurrence of *ehxA* and other virulence genes

The intimin gene *eae* and three putative pO157-derived virulence genes (*toxB*, *katP*, and *espP*) were screened among the 138 *ehxA*-positive non-O157 STEC isolates. In total, only 15 (10.9%) isolates harboured *eae*, among which two (1.7%) were placed into *ehxA* group II and 13 (81.2%) into group II (Table 2). Among the 13 isolates that were both *eae* and *ehxA*-positive, eight were from diarrhoeal patients. These results showed that *ehxA* group II strains were mostly *eae*-positive and clinically related, while strains in *ehxA* group I and group III might be *eae*-negative (*P < *0.05). In addition, *espP* was present in 72 (52.2%) isolates, but only eight (5.8%) and five (3.6%) isolates were *katP* and *toxB* positive.

### Haemolytic activity on SHIBAM plates

Among the 138 *ehxA*-positive non-O157 STEC strains, 93 (67.4%) strains showed haemolytic activity against washed sheep red blood cells. The haemolysis ability varied among the strains. Fifty-five strains showed relatively clear transparent zones on the SHIBAM plates, while only narrow haemolytic zones were observed for 38 strains (Table S2). Haemolytic strains in *ehxA* groups I and II were 69.4% (84/121) and 56.3% (9/16), respectively. The *ehxA* group III strain did not show haemolytic activity. No statistical significance was observed for the correction between haemolytic activity and *ehxA* groups (*P* > 0.05).

### Plasmid incompatibility (Inc)/replicon (Rep) profiles in *ehxA*-positive non-O157 STEC strains

Eighteen different plasmid replicons were screened by PCR, including HI1, HI2, I1-Ig, X, L/M, N, FIA, FIB, W, Y, P, FIC, T, A/C, FIIA, K, F, and B/O. Five types of replicons (I1-Ig, FIB, K, F, and B/O) were detected (Table S2). Among them, 123 (89.1%) and 107 (77.5%) strains were positive for FIB and F replicons, respectively. K and B/O replicons were present in 24 (17.4%) and 21 (15.2%) strains, respectively. Only one strain contained the I1-Ig replicon. Most strains (82.6%) harboured two or more replicons; however, four strains did not harbour any replicons.

All five plasmid replicons were observed in *ehxA* group I strains and three plasmid replicons were detected in group II. The only strain in group III contained replicons FIB and F.

## Discussion

STEC related virulence factors, such as Shiga toxin and intimin, have been well investigated worldwide, especially for O157:H7 and other predominant serotypes^20,21^. Nevertheless, enterohaemorrhagic *E. coli* haemolysin (Ehx) is an increasingly recognized putative virulence factor^22^. In the present study, we systematically investigated the prevalence of the *ehxA* gene in non-O157 STEC strains isolated from a variety of sources and different geographical locations in China. Our study showed that the *ehxA* gene was present in 31.8% (138/434) of the non-O157 STEC strains, which was lower than that of a previous report (69.2%)^23^, indicating that the prevalence of *ehxA* among non-O157 STEC might vary in different sources or geographical locations.

The *ehxA* gene is relatively conserved in STEC strains, and six *ehxA* subtypes have been reported based on PCR-RFLP subtyping methods^23^. *ehxA* subtypes may provide information on the epidemiology and evolution of *E. coli*; however, the traditional PCR-RFLP subtyping method is time-consuming and laborious. Furthermore, cross-reactions sometimes occurred and appeared as ghost bands on gel electrophoresis, especially for those strains with high sequence similarity, which call for additional stringency to differentiate them. Notably, PCR-RFLP protocols have generated contradictory results for the subtyping of some toxins, for example, Shiga toxin subtypes^7^. Here, a sequence-based phylogenetic approach was used for *ehxA* subtyping. By comparing the complete *ehxA* sequences of 138 *ehxA*-positive STEC isolates from in this study and those assigned to six existing subtypes^23^, three clear phylogenetic groups were formed. Additionally, the alignment of all known subtypes allowed us to evaluate the PCR-RFLP subtyping method and identify possible misinterpretations of PCR-RFLP results, as this method has never really been validated on *ehxA* against a representative number of strains. Our results showed that the phylogenic relatedness of the three *ehxA* groups assigned in this study were in agreement with that of the A-F subtypes reported previously^23^. *ehxA* group I comprised *ehxA* sequences that were assigned as the A and E subtypes; group II comprised sequences assigned as *ehxA* subtype B, C, and F, which were found to show a close relationship; while group III contained only the *ehxA* D subtype, which formed the most divergent subdivision and was well separated from the other groups/subtypes (Fig. 1). It was deduced that group III or subtype D strains might carry an *ehxA*-containing plasmid that is different from the other subtypes^24^.

The current study demonstrated a correlation between *ehxA* groups and their sources, which was in agreement with a previous study^10^. Healthy carrier strains, and most animal (96.2%) and food-derived strains (80.0%) fell into *ehxA* group I, where *toxB* and *espP* gene were detected in five (4.1%) and 60 (49.6%) isolates, and the serotypes were diverse. Remarkably, all strains from diarrhoeal patients belonged to group II, in which most strains harboured *eae* (81.3%), *katP* (50.0%), and *espP* (50.0%), and high pathogenic serotypes: i.e, O26:H11, O111:H8, and O103:H25 were observed. It can be inferred that *ehxA* group II strains showed higher pathogenic potential compared with those in group I, while strains in group III were less associated with human illness. It should be noted that 20.0% of food-derived strain and 2.7% of the animal strains were classified into group II, further enhancing our previous finding that some of the food- and animal-derived strains showed high pathogenic potential; thus, humans might be infected by the consumption of foods or contamination from animals^19^.

The *ehxA* positive strains have been demonstrated to have an enterohaemolytic phenotype *in vitro* on washed sheep blood agar^10^. In this study, 93 (67.4%) strains showed enterohaemolytic activity, which was less than that described in a previous study, where 276 (82.8%) of *ehxA*-positive STEC strains showed enterohaemolytic capacity^23^. It could be inferred that some non-O157 STECs showed lower tendency to cause haemolysis, especially among those less predominant serotypes. No obvious correlation between haemolytic capacity and *ehxA* groups was observed. By contrast, the absence of an enterohaemolytic phenotype indicated that the precise conditions for the regulation and optimum expression of enterohaemolysin among the non-O157 STEC strains remains to be further determined^22^.

*E. coli* possess a variety of plasmids, and many plasmids are associated with pathogenicity. Plasmids are defined as incompatibility groups when they have the same replication mechanisms^25,26^. The *ehxA* gene is located on the F-like plasmid pO157 in *E. coli* O157:H7^18^. We tested eighteen plasmid incompatibility groups, and five groups were detected in 138 *ehxA*-positive non-O157 STEC. FIB and F were the major plasmid incompatibility groups detected in this study. Furthermore, plasmid pO157 contains others genes, such as *katP*, *espP*, and *toxB*^18^. In a previous report, plasmids resembling pO157 were found in non-O157 STEC strains that carried *ehxA*, *katP* and *espP* genes in less than 50% of strains^27^. In this study, only 3.6% and 5.8% of strains harboured the *toxB* and *katP* genes, respectively, while *espP* was positive in 52.2% of isolates. These results implied a marked genetic diversity of *ehxA* containing plasmids in non-O157 STEC strains, which requires further analysis.

In conclusion, this study systematically investigated the prevalence of the enterohaemolysin gene *ehxA* in non-O157 STEC strains from a variety of sources collected from different geographical locations in China. A sequence-based phylogenetic approach was used for *ehxA* genotyping, based on a wide variety of strains. In total, 138 *ehxA*-positive non-O157 STEC strains were identified from among 434 strains, which were phylogenetically clustered into three groups (I, II, and III). Correlations between the *ehxA* groups and the sources, serotypes, and virulent gene profiles were observed. The *ehxA* group II non-O157 STEC strains were mostly diarrhoeal patient-derived and may demonstrate a higher pathogenic potential compared with group I and group III strains. These results are of great importance in modern strain typing and in epidemiological surveillance of non-O157 STEC infections.

## Methods

### Bacterial strains

A total of 434 non-O157 STEC strains representing 95 different O-serogroups were collected during April 2009 to August 2016 in ten geographical regions in China (Tables 1 and S1). Most strains were obtained and reported in our previous investigations^19,28–31^. Only a small number of strains were isolated from samples collected by local centres for disease control and prevention. These strains were isolated from various sources, including cattle/cow, goat, pig, yak, marmot, pika, antelope, food, patients with diarrhoea, and healthy carriers. All strains were confirmed to be STEC using previously described methods^19^.

### Serotyping and detection of virulence genes

The O:H serotyping of each strain was determined using previously described methods^29^. The enterohaemolysin gene *ehxA* of all 434 non-O157 STEC strains, and *eae*, *katP*, *espP* and *toxB* genes of all *ehxA*-positive strains were determined using PCR with primer described previously^30^.

### Haemolysis test

All *ehxA*-positive STEC strains were inoculated overnight on Luria-Bertani (LB) agar (Oxoid, UK). A single fresh colony was picked and inoculated onto SHIBAM plates and incubated aerobically at 37 °C. SHIBAM plates are brain-heart infusion medium supplemented with 10 mM CaCl_2_, 5% phosphate-buffered saline (PBS)-washed defibrinated sheep blood, and 0.5 μg/ml mitomycin C^32^. Hemolysis was observed after 6 and 24 h, as described previously^10^. Each strain was tested in duplicate.

### Sequencing of the complete *ehxA* gene

The complete *ehxA* gene was obtained by PCR using previously described methods^10,23^. Two other pairs of primers designed in this study were used for sequencing: *ehxA* F-W1F (5′-TGGGCTGGATGTTGTCTC-3′) and *ehxA* R-W1R (5′-TTCCACTACCACCAAATAAC-3′); *ehxA* R-W1F-low (5′-GTTATAACAGATAAAGATGGTCG-3′) and *ehxA* F-W1R-low (5′-CTGGTTTGCAATCGCTGTATCAT-3′). The PCR product was purified using a QIAquick PCR Purification kit (Qiagen, Hilden, Germany) and sequenced using the BigDye™ Terminator V3.1 Cycle Sequencing kit (Applied Biosystems, USA).

### Phylogenetic analysis

The sequenced ~2997-bp *ehxA* was assembled using SeqMan II (DNASTAR Inc., USA). The representatives of reference *ehxA* sequences of six *ehxA* subtypes A to F were downloaded from GenBank^10,24^. The *ehxA* sequences obtained in this study and the reference sequences were aligned using MEGA 7 software (www.megasoftware.net)^33^. Phylogenetic trees were constructed with two algorithms, neighbour-joining (NJ), and maximum-likelihood (ML), using MEGA 7. The stability was estimated by bootstrap analysis (1000 replications), and genetic distances were calculated by the maximum composite likelihood method. A sequence was designated to a specific *ehxA* group based on its phylogenetic placement.

### Whole genome sequencing

Forty-two *ehxA*-positive strains representing diverse sources, *stx* subtypes, and virulence profiles were selected for whole genome sequencing (WGS). For each strain, a 500–2000 bp library was constructed and then sequenced on an Illumina HiSeq. 2500-PE125 (Illumina, San Diego, CA, USA) to produce 150 bp paired-end reads. These qualified reads were then assembled into scaffolds using the program SOAP *de novo* (http://soap.genomics.org.cn/soapdenovo.html). Open reading frames (ORFs) were identified and annotated using the Artemis program (www.sanger.ac.uk) and homology searches were performed against several databases, including GenBank (www.ncbi.nlm.nih.gov/GenBank)^34^. The *ehxCABD* gene cluster was extracted from the draft genome sequences using a perl script. Three genes, *katP*, *toxB* and *espP*, were searched against the 42 genome sequences using BLAST.

### PCR-based replicon typing

Eighteen pairs of primers were used to determine the plasmid incompatibility (Inc) groups in all *ehxA*-positive strains, as described previously^25^. The assays were performed in five multiplex-PCR and three simplex-PCR reactions. PCR conditions are as follows: initial denaturation of 94 °C for 5 min; followed by 30 cycles of 94 °C for 1 min, 60 °C for 30 s and 72 °C for 1 min; and a final extension at 72 °C for 5 min, with the exception of the IncF-simplex PCR, where the annealing temperature was set at 52 °C.

### Statistical analysis

The correlations between *ehxA* groups and STEC origins, serogroups, haemolytic ability, or virulence genes were analysed using Fisher’s exact test. SAS^®^ 9.3 (SAS Institute Inc., USA) was used to perform the calculations. A *P* value < 0.05 was considered significant statistically.

### Nucleotide sequence accession numbers

Thirty-six complete *ehxA* sequences obtained in this study were submitted to GenBank under the accession numbers MF802290–MF802325.

## Electronic supplementary material

Supplementary Information
