# Supplementary material for: Genetic diversity of the enterohaemolysin gene (ehxA) in non-O157 Shiga toxin-producing Escherichia coli strains in China
Source: Sci Rep. 2018 Mar 9;8:4233. doi: 10.1038/s41598-018-22699-7 (PMC5844952; doi:10.1038/s41598-018-22699-7)
Supplement: Supplementary file 1 — Supplementary Information [file 41598_2018_22699_MOESM1_ESM.pdf]

Supplementary Information

Genetic diversity of the enterohaemolysin gene (*ehxA*) in non-O157 Shiga toxin-producing *Escherichia coli* strains in China

Shanshan Fu [1], Xiangning Bai [1], Ruyue Fan [1], Hui Sun [1], Yanmei Xu [1] and Yanwen Xiong [1,2*]

[1] State Key Laboratory of Infectious Disease Prevention and Control, National Institute for Communicable Disease Control and Prevention, Chinese Center for Disease Control and Prevention, Changping, Beijing, China

[2] Collaborative Innovation Center for Diagnosis and Treatment of Infectious Diseases, Hangzhou, Zhejiang Province, China

* Corresponding author. Tel.: +8610 58900749; Fax: +8610 58900748

E-mail address: xiongyanwen@icdc.cn

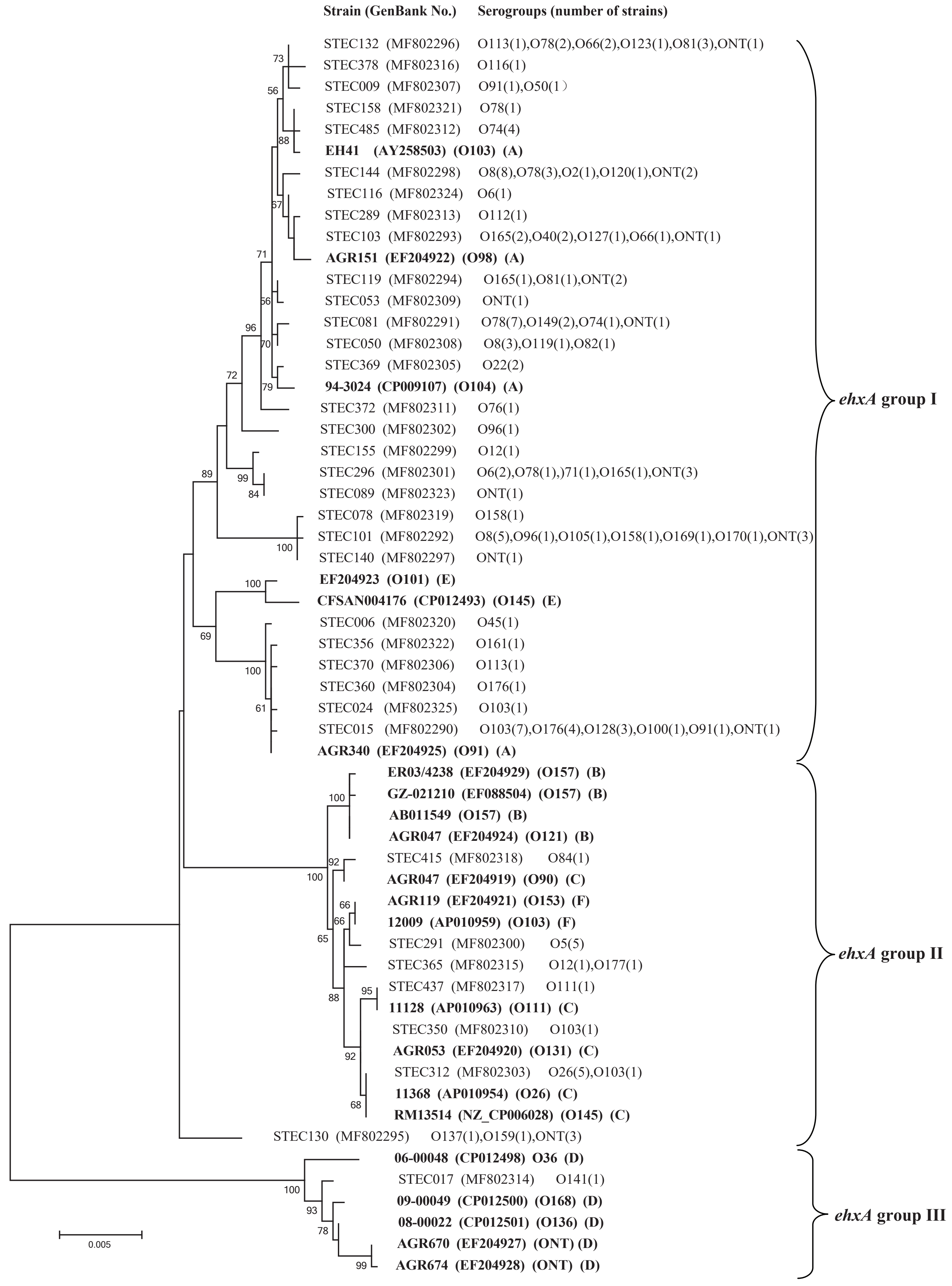

Figure S1. Phylogentic relationships of *ehxA* sequences based on the maximum-likelihood method.

Thirty-six unique *ehxA* sequences were obtained in this study. The serogroups for each *ehxA* genotype (representative strain) are given. Twenty-two sequences of six *ehxA* PCR-RFLP subtypes A to F downloaded from GenBank are indicated in bold. Bootstrap values > 50% are shown at the branch points.

Table S1. Characteristics of 434 non-O157 STEC strains used in this study

Strain	Origin	Location	Year	O serogroup	H type	Virulence genes			
						stx1	*stx2*	*eae*	*ehxA*
ST003	Goat	Henan	2009	O45	H2	*stx1c*	*stx2d*	-	-
ST004	Goat	Henan	2009	O118	H16	-	*stx2d*	-	-
ST005	Goat	Henan	2009	O39	H19	-	*stx2e*	-	-
ST006	Goat	Henan	2009	O45	H2	*stx1c*	*stx2d*	-	+
ST007	Goat	Henan	2009	O91	H14	*stx1c*	*stx2d*	-	+
ST008	Goat	Henan	2009	O21	H25	-	*stx2d*	-	-
ST009	Goat	Henan	2009	O91	H14	*stx1c*	*stx2d*	-	+
ST010	Goat	Henan	2009	O110	H16	*stx1a*	*stx2d*	-	-
ST011	Goat	Henan	2009	O103	H8	*stx1c*	-	-	+
ST012	Goat	Henan	2009	O21	H25	*stx1a*	-	-	-
ST013	Goat	Henan	2009	O21	H25	*stx1a*	-	-	-
ST014	Goat	Henan	2009	O21	H25	*stx1a*	-	-	-
ST015	Goat	Henan	2009	O103	H8	*stx1c*	-	-	+
ST016	Goat	Henan	2009	O103	H8	*stx1c*	-	-	+
ST017	Goat	Henan	2009	O141ac	H25	-	*stx2g*	-	+
ST018	Goat	Henan	2009	O15	H21	*stx1a*	-	-	-
ST019	Goat	Henan	2009	O12	H21	*stx1a*	-	-	-
ST020	Goat	Henan	2009	O12	H21	*stx1a*	-	-	-
ST021	Goat	Henan	2009	O21	H25	*stx1a*	-	-	-
ST022	Goat	Henan	2009	O21	H25	*stx1a*	-	-	-
ST023	Goat	Henan	2009	O103	H8	*stx1c*	-	-	+
ST024	Goat	Henan	2009	O103	H8	*stx1c*	-	-	+
ST025	Goat	Henan	2009	O103	H8	*stx1c*	-	-	+
ST026	Goat	Henan	2009	O103	H8	*stx1c*	-	-	+
ST027	Goat	Henan	2009	O103	H8	*stx1c*	-	-	+
ST028	Goat	Henan	2009	OUT	H21	*stx1c*	-	-	-
ST029	Goat	Henan	2009	O15	H21	*stx1c*	-	-	-
ST030	Goat	Henan	2009	OUT	H21	*stx1a*	-	-	-
ST031	Diarrheal patient	Henan	2011	O107	H7	*stx1a*	-	-	-
ST032	Diarrheal patient	Henan	2011	O107	H7	*stx1a*	-	-	-
ST033	Beef cattle	Heilongjiang	2009	O113	H19	*stx1a*	*stx2a*	-	+
ST036	Yak	Qinghai	2012	NT	H21	-	*stx2d*	-	+
ST037	Yak	Qinghai	2012	NT	H44	*stx1a*	-	-	+
ST038	Yak	Qinghai	2012	O8	H9	*stx1a*	*stx2d*	-	+
ST039	Yak	Qinghai	2012	NT	H7	*stx1a*	-	-	+
ST040	Yak	Qinghai	2012	O6	H21	*stx1a*	-	-	+
ST041	Yak	Qinghai	2012	O6	H21	*stx1a*	-	-	-

ST042	Yak	Qinghai	2012	O78	H45	-	*stx2g*	-	+
ST043	Yak	Qinghai	2012	O8	H9	*stx1a*	*stx2d*	-	+
ST044	Yak	Qinghai	2012	O8	H16	-	*stx2b*	-	-
ST045	Yak	Qinghai	2012	O8	H9	*stx1a*	*stx2d*	-	+
ST046	Yak	Qinghai	2012	O6	NT	*stx1a*	-	-	+
ST047-1	Yak	Qinghai	2012	O165	H21	*stx1a*	-	-	+
ST047-2	Yak	Qinghai	2012	O78	H45	-	*stx2g*	-	-
ST048	Yak	Qinghai	2012	O78	H45	-	*stx2g*	-	-
ST049	Yak	Qinghai	2012	NT	NT	-	*stx2c*	-	+
ST050	Yak	Qinghai	2012	O8	H19	*stx1a*	-	-	+
ST051	Yak	Qinghai	2012	NT	H21	*stx1a*	-	-	+
ST052	Yak	Qinghai	2012	NT	H21	*stx1a*	-	-	+
ST053	Yak	Qinghai	2012	NT	NT	*stx1a*	*stx2a+stx2b*	-	+
ST054	Yak	Qinghai	2012	NT	H8	*stx1a*	*stx2a+stx2b*	-	+
ST055	Yak	Qinghai	2012	O66	H8	*stx1a*	-	-	+
ST056	Yak	Qinghai	2012	O78	NT	-	*stx2a*	+	+
ST057	Yak	Qinghai	2012	O78	H21	-	*stx2c*	-	+
ST058	Yak	Qinghai	2012	O22	H8	-	*stx2b*	-	-
ST059	Yak	Qinghai	2012	O12/O78	H44	-	*stx2d*	-	-
ST060	Yak	Qinghai	2012	O117	H21	-	*stx2b*	-	-
ST061	Yak	Qinghai	2012	O78	H45	-	*stx2g*	-	-
ST062	Yak	Qinghai	2012	O8	H45	-	*stx2g*	-	-
ST063	Yak	Qinghai	2012	O8	H19	*stx1a*	-	-	+
ST064	Yak	Qinghai	2012	O8	H19	*stx1a*	-	-	+
ST065	Yak	Qinghai	2012	O8	H9	*stx1a*	*stx2d*	-	+
ST066	Yak	Qinghai	2012	O8	H9	*stx1a*	*stx2d*	-	+
ST067	Yak	Qinghai	2012	O8	H16	-	*stx2b*	-	-
ST068	Yak	Qinghai	2012	O78	H21	-	*stx2a*	+	+
ST069	Yak	Qinghai	2012	O52	H2	-	*stx2b*	-	-
ST070	Yak	Qinghai	2012	O78	H45	-	*stx2g*	-	-
ST071	Yak	Qinghai	2012	O8	H9	*stx1a*	*stx2d*	-	+
ST072	Yak	Qinghai	2012	O8	H9	*stx1a*	*stx2d*	-	+
ST073	Yak	Qinghai	2012	O8	H9	*stx1a*	*stx2d*	-	+
ST074	Yak	Qinghai	2012	O117	H21	-	*stx2b*	-	-
ST075	Yak	Qinghai	2012	O66	H21	-	*stx2a+stx2b*	-	+
ST076	Yak	Qinghai	2012	NT	H8	-	*stx2b*	-	+
ST077	Yak	Qinghai	2012	O158	H8	-	*stx2b*	-	+
ST078	Yak	Qinghai	2012	O158	H8	-	*stx2b*	-	+
ST079	Yak	Qinghai	2012	O2	H45	*stx1a*	-	-	-
ST080	Yak	Qinghai	2012	O2	H45	*stx1a*	-	-	-
ST081	Yak	Qinghai	2012	O78	H8	-	*stx2a*	-	+
ST082	Yak	Qinghai	2012	O78	H8	-	*stx2a*	-	+
ST083	Yak	Qinghai	2012	O2	H45	*stx1a*	-	-	-

ST084	Yak	Qinghai	2012	O66	H21	-	*stx2a+stx2b*	-	+
ST085	Yak	Qinghai	2012	O8	H16	-	*stx2b*	-	-
ST086	Yak	Qinghai	2012	O8	H45	-	*stx2b*	-	-
ST087	Yak	Qinghai	2012	O8	H45	-	*stx2b*	-	-
ST088	Yak	Qinghai	2012	O22	H8	-	*stx2b*	-	-
ST089	Yak	Qinghai	2012	NT	H21	*stx1a*	-	-	+
ST090	Yak	Qinghai	2012	O22	H8	-	*stx2b*	-	-
ST091	Yak	Qinghai	2012	NT	H21	*stx1a*	-	-	+
ST092	Yak	Qinghai	2012	NT	H8	-	*stx2b*	-	+
ST093	Yak	Qinghai	2012	O2	H45	*stx1a*	-	-	-
ST094	Yak	Qinghai	2012	O137	H21	*stx1a*	*stx2a*	-	+
ST095	Yak	Qinghai	2012	NT	H8	*stx1a*	-	-	+
ST096	Yak	Qinghai	2012	O127	H8	*stx1a*	*stx2d*	-	+
ST097	Yak	Qinghai	2012	O2	H45	*stx1a*	-	-	-
ST098	Yak	Qinghai	2012	O117	H21	-	*stx2b*	-	-
ST099	Yak	Qinghai	2012	O117	H21	-	*stx2b*	-	-
ST100	Yak	Qinghai	2012	NT	H8	*stx1a*	*stx2b*	-	+
ST101	Yak	Qinghai	2012	O8	H2	-	*stx2d*	-	+
ST102	Yak	Qinghai	2012	O165	H8	*stx1a*	-	-	+
ST103	Yak	Qinghai	2012	O165	H8	*stx1a*	-	-	+
ST104	Yak	Qinghai	2012	O8	H16	-	*stx2b*	-	-
ST105	Yak	Qinghai	2012	O8	H16	-	*stx2b*	-	-
ST106	Yak	Qinghai	2012	O117	H2	-	*stx2b*	-	-
ST107	Yak	Qinghai	2012	O117	H2	-	*stx2b*	-	-
ST108	Yak	Qinghai	2012	O117	H2	-	*stx2b*	-	-
ST109	Yak	Qinghai	2012	O117	H2	-	*stx2b*	-	-
ST110	Yak	Qinghai	2012	O8	H16	-	*stx2b*	-	-
ST111	Yak	Qinghai	2012	O8	H16	-	*stx2b*	-	-
ST112	Yak	Qinghai	2012	O117	H21	-	*stx2b*	-	-
ST113	Yak	Qinghai	2012	O117	H21	-	*stx2b*	-	-
ST114	Yak	Qinghai	2012	O117	H21	-	*stx2b*	-	-
ST115	Yak	Qinghai	2012	O6	H14	-	*stx2b*	-	+
ST116	Yak	Qinghai	2012	O6	H14	-	*stx2b*	-	+
ST117	Yak	Qinghai	2012	O158	H16	-	*stx2b*	-	-
ST118	Yak	Qinghai	2012	O8	H16	-	*stx2b*	-	-
ST119	Yak	Qinghai	2012	NT	H8	*stx1a*	*stx2d*	-	+
ST120	Yak	Qinghai	2012	O165	H8	*stx1a*	*stx2d*	-	+
ST121	Yak	Qinghai	2012	O117	H21	-	*stx2b*	-	-
ST122	Yak	Qinghai	2012	NT	H40	-	*stx2b*	-	-
ST123	Yak	Qinghai	2012	O117	H21	-	*stx2b*	-	-
ST124	Yak	Qinghai	2012	O117	H21	-	*stx2b*	-	-
ST125	Yak	Qinghai	2012	O8	H16	-	*stx2b*	-	-
ST126	Yak	Qinghai	2012	O117	H21	-	*stx2b*	-	-

ST127	Yak	Qinghai	2012	O78	H8	*stx1a*	*stx2d*	-	+
ST128	Yak	Qinghai	2012	O78	H8	*stx1a*	*stx2d*	-	+
ST129	Yak	Qinghai	2012	NT	H21	*stx1a*	*stx2d*	-	+
ST130	Yak	Qinghai	2012	NT	H21	*stx1a*	*stx2d*	-	+
ST131	Yak	Qinghai	2012	NT	H44	*stx1a*	-	-	+
ST132	Yak	Qinghai	2012	O123	H8	*stx1a*	*stx2b*	-	+
ST133	Yak	Qinghai	2012	O8	H16	-	*stx2b*	-	-
ST134	Yak	Qinghai	2012	O8	H16	-	*stx2b*	-	-
ST135	Yak	Qinghai	2012	O8	H16	-	*stx2b*	-	-
ST136	Yak	Qinghai	2012	O8	H16	-	*stx2b*	-	-
ST137	Yak	Qinghai	2012	O8	H16	-	*stx2b*	-	-
ST138	Yak	Qinghai	2012	O149	H45	-	*stx2d*	-	+
ST139	Yak	Qinghai	2012	O149	H45	-	*stx2d*	-	+
ST140	Yak	Qinghai	2012	NT	H8	-	*stx2a*	-	+
ST141	Yak	Qinghai	2012	NT	H8	-	*stx2a*	-	+
ST142	Yak	Qinghai	2012	O2	H45	*stx1a*	-	-	-
ST143	Yak	Qinghai	2012	O2	H45	*stx1a*	-	-	-
ST144	Yak	Qinghai	2012	O78	H45	-	*stx2a+2c*	-	+
ST145	Yak	Qinghai	2012	O78	H21	-	*stx2a+2c*	-	+
ST146	Yak	Qinghai	2012	O2	H21	-	*stx2a+2c*	-	+
ST147	Yak	Qinghai	2012	O2	H45	*stx1a*	-	-	-
ST148	Yak	Qinghai	2012	O22	H8	-	*stx2b*	-	-
ST149	Yak	Qinghai	2012	O2	H45	*stx1a*	-	-	-
ST150	Yak	Qinghai	2012	O2	H45	*stx1a*	-	-	-
ST151	Yak	Qinghai	2012	O2	H45	*stx1a*	-	-	-
ST152	Yak	Qinghai	2012	O78	H8	-	*stx2a*	-	+
ST153	Yak	Qinghai	2012	O78	H8	-	*stx2a*	-	+
ST154	Yak	Qinghai	2012	O78	H44	-	*stx2d*	-	-
ST155	Yak	Qinghai	2012	O12	H12	*stx1a*	-	-	+
ST156	Yak	Qinghai	2012	O78	H8	-	*stx2a*	-	+
ST157	Yak	Qinghai	2012	O2	H45	*stx1a*	-	-	-
ST158	Yak	Qinghai	2012	O78	H8	-	*stx2a+2c*	-	+
ST159	Yak	Qinghai	2012	O2	H45	*stx1a*	-	-	-
ST160	Yak	Qinghai	2012	O2	H45	*stx1a*	-	-	-
ST161	Yak	Qinghai	2012	O52	H2	-	*stx2b*	-	-
ST162	Yak	Qinghai	2012	O52	H2	-	*stx2b*	-	-
ST163	Pig	Chongqing	2012	O2	H32	-	*stx2e*	-	-
ST164	Pig	Chongqing	2012	O2	H32	-	*stx2e*	-	-
ST165	Pig	Chongqing	2012	O2	H32	-	*stx2e*	-	-
ST166	Pig	Chongqing	2012	O2	H32	-	*stx2e*	-	-
ST167	Pig	Chongqing	2012	O2	H32	-	*stx2e*	-	-
ST168	Pig	Chongqing	2012	O2	H32	-	*stx2e*	-	-
ST169	Pig	Chongqing	2012	O2	H32	-	*stx2e*	-	-

ST170	Pig	Chongqing	2012	O2	H32	-	*stx2e*	-	-
ST171	Pig	Chongqing	2012	O116	H11	-	*stx2e*	-	-
ST172	Pig	Chongqing	2012	O2	H32	-	*stx2e*	-	-
ST173	Pig	Chongqing	2012	O20	H30	-	*stx2e*	-	-
ST174	Pig	Chongqing	2012	O20	H26	-	*stx2e*	-	-
ST175	Pig	Chongqing	2012	O20	H30	-	*stx2e*	-	-
ST176	Pig	Chongqing	2012	O76	H25	-	*stx2e*	-	-
ST177	Pig	Chongqing	2012	O20	H26	-	*stx2e*	-	-
ST178	Pig	Chongqing	2012	O20	H30	-	*stx2e*	-	-
ST179	Pig	Chongqing	2012	O20	H30	-	*stx2e*	-	-
ST180	Pig	Chongqing	2012	ONT	H30	-	*stx2e*	-	-
ST181	Pig	Chongqing	2012	ONT	H30	-	*stx2e*	-	-
ST182	Pig	Chongqing	2012	O114	H30	-	*stx2e*	-	-
ST183	Pig	Chongqing	2012	O65	H30	-	*stx2e*	-	-
ST184	Pig	Chongqing	2012	O100	H20	-	*stx2e*	-	-
ST185	Pig	Chongqing	2012	O100	H20	-	*stx2e*	-	-
ST186	Pig	Chongqing	2012	O20	H30	-	*stx2e*	-	-
ST187	Pig	Chongqing	2012	O20	H30	-	*stx2e*	-	-
ST188	Pig	Beijing	2012	ONT	H21	-	*stx2e*	-	-
ST189	Pig	Beijing	2012	ONT	H21	-	*stx2e*	-	-
ST190	Pig	Beijing	2012	ONT	H33	-	*stx2e*	-	-
ST191	Pig	Beijing	2012	ONT	H30	-	*stx2e*	-	-
ST192	Pig	Beijing	2012	O2	H32	-	*stx2e*	-	-
ST193	Pig	Beijing	2012	O2	H32	-	*stx2e*	-	-
ST194	Pig	Beijing	2012	ONT	H30	-	*stx2e*	-	-
ST195	Pig	Beijing	2012	ONT	H30	-	*stx2e*	-	-
ST196	Pig	Beijing	2012	O2	H30	-	*stx2e*	-	-
ST197	Pig	Beijing	2012	O172	H30	-	*stx2e*	-	-
ST198	Pig	Beijing	2012	O172	H30	-	*stx2e*	-	-
ST199	Pig	Beijing	2012	O20	H30	-	*stx2e*	-	-
ST200	Pig	Beijing	2012	O20	H30	-	*stx2e*	-	-
ST201	Pig	Beijing	2012	O20	H30	-	*stx2e*	-	-
ST202	Pig	Beijing	2012	O20	H30	-	*stx2e*	-	-
ST203	Pig	Beijing	2012	ONT	H30	-	*stx2e*	-	-
ST204	Pig	Beijing	2012	O20	H30	-	*stx2e*	-	-
ST205	Pig	Beijing	2012	O20	H30	-	*stx2e*	-	-
ST206	Pig	Beijing	2012	O20	H30	-	*stx2e*	-	-
ST207	Pig	Beijing	2012	O20	H30	-	*stx2e*	-	-
ST208	Pig	Beijing	2012	ONT	H30	-	*stx2e*	-	-
ST209	Pig	Beijing	2012	ONT	H30	-	*stx2e*	-	-
ST210	Pig	Beijing	2012	O7	H30	-	*stx2e*	-	-
ST211	Pig	Beijing	2012	ONT	H19	-	*stx2e*	-	-
ST212	Pig	Beijing	2012	ONT	H19	-	*stx2e*	-	-

ST213	Pig	Beijing	2012	ONT	H19	-	*stx2e*	-	-
ST214	Pig	Beijing	2012	O100	H30	-	*stx2e*	-	-
ST215	Pig	Beijing	2012	O20	H30	-	*stx2e*	-	-
ST216	Pig	Beijing	2012	O20	H30	-	*stx2e*	-	-
ST217	Pig	Beijing	2012	O20	H30	-	*stx2e*	-	-
ST218	Pig	Beijing	2012	O20	H30	-	*stx2e*	-	-
ST219	Pig	Beijing	2012	O114	H30	-	*stx2e*	-	-
ST220	Pig	Beijing	2012	O7	H42	-	*stx2e*	-	-
ST221	Pig	Beijing	2012	O172	H30	-	*stx2e*	-	-
ST222	Pig	Beijing	2012	ONT	H30	-	*stx2e*	-	-
ST223	Pig	Beijing	2012	ONT	H30	-	*stx2e*	-	-
ST224	Pig	Beijing	2012	ONT	H30	-	*stx2e*	-	-
ST225	Pig	Beijing	2012	ONT	H30	-	*stx2e*	-	-
ST226	Pig	Beijing	2012	ONT	H30	-	*stx2e*	-	-
ST227	Pig	Beijing	2012	ONT	H30	-	*stx2e*	-	-
ST228	Pig	Beijing	2012	ONT	H30	-	*stx2e*	-	-
ST229	Pig	Beijing	2012	ONT	H20	-	*stx2e*	-	-
ST230	Pig	Beijing	2012	ONT	H20	-	*stx2e*	+	-
ST231	Pig	Beijing	2012	ONT	ONT	-	*stx2e*	-	-
ST232	Pig	Beijing	2012	ONT	H20	-	*stx2e*	-	-
ST233	Pig	Beijing	2012	O65	H30	-	*stx2e*	-	-
ST234	Pig	Beijing	2012	O142	H27	-	*stx2e*	-	-
ST235	Pig	Beijing	2012	O86	H11	-	*stx2e*	-	-
ST236	Pig	Beijing	2012	ONT	H30	-	*stx2e*	-	-
ST237	Pig	Beijing	2012	O20	H30	-	*stx2e*	-	-
ST238	Pig	Beijing	2012	O9	H30	-	*stx2e*	-	-
ST239	Pig	Beijing	2012	ONT	H30	-	*stx2e*	-	-
ST240	Pig	Beijing	2012	O143	H38	-	*stx2e*	-	-
ST241	Pig	Beijing	2012	O143	H38	-	*stx2e*	-	-
ST242	Pig	Beijing	2012	ONT	H17	-	*stx2e*	-	-
ST243	Pig	Beijing	2012	ONT	H17	-	*stx2e*	-	-
ST244	Pig	Beijing	2012	O5(O70)	H30	-	*stx2e*	-	-
ST245	Pig	Beijing	2012	O5(O70)	H30	-	*stx2e*	+	-
ST246	Pig	Beijing	2012	O65	H30	-	*stx2e*	-	-
ST247	Pig	Beijing	2012	O20	H30	-	*stx2e*	-	-
ST248	Pig	Beijing	2012	ONT	H30	-	*stx2e*	-	-
ST249	Pig	Beijing	2012	ONT	H30	-	*stx2e*	-	-
ST250	Pig	Beijing	2012	O20	H30	-	*stx2e*	-	-
ST251	Pig	Beijing	2012	ONT	H30	-	*stx2e*	-	-
ST252	Pig	Beijing	2012	O9	H30	-	*stx2e*	-	-
ST253	Pig	Beijing	2012	O100	H30	-	*stx2e*	-	-
ST254	Pig	Beijing	2012	O5(O70)	H30	-	*stx2e*	-	-
ST255	Pig	Beijing	2012	O86	H11	-	*stx2e*	-	-

ST256	Pig	Beijing	2012	O9	H30	-	*stx2e*	-	-
ST257	Pig	Beijing	2012	O9	H30	-	*stx2e*	-	-
ST258	Pig	Beijing	2012	O20	H30	-	*stx2e*	-	-
ST259	Pig	Beijing	2012	O71	H30	-	*stx2e*	-	-
ST260	Pig	Beijing	2012	O116	H30	-	*stx2e*	-	-
ST261	Pig	Beijing	2012	O65	H30	-	*stx2e*	-	-
ST262	Pig	Beijing	2012	O65	H30	-	*stx2e*	-	-
ST263	Pig	Beijing	2012	O65	H30	-	*stx2e*	-	-
ST264	Pig	Beijing	2012	ONT	H30	-	*stx2e*	-	-
ST265	Pig	Beijing	2012	O100	H30	-	*stx2e*	-	-
ST266	Pig	Beijing	2012	O143	H38	-	*stx2e*	-	-
ST267	Pig	Beijing	2012	ONT	H19	-	*stx2e*	-	-
ST268	Pig	Harbin	2012	O100	H30	-	*stx2*	-	-
ST269	Pig	Harbin	2012	O173	H21	-	*stx2*	-	-
ST270	Pig	Harbin	2012	O2 (O50)	H30	-	*stx2*	-	-
ST271	Pig	Harbin	2012	O2 (O50)	H30	-	*stx2*	-	-
ST272	Pig	Harbin	2012	O2 (O50)	H30	-	*stx2*	-	-
ST273	Pig	Harbin	2012	O2 (O50)	H32	-	*stx2*	-	-
ST274	Pig	Harbin	2012	O2 (O50)	H32	-	*stx2*	-	-
ST275	Pig	Harbin	2012	O168	H38	-	*stx2*	-	-
ST276	Beef cattle	Harbin	2012	ONT	H30	-	*stx2g*	-	-
ST277	Beef cattle	Harbin	2012	O71	H30	-	*stx2*	-	-
ST278	Beef cattle	Harbin	2012	ONT	H30	-	*stx2g*	-	-
ST280	Beef cattle	Harbin	2012	ONT	H30	-	*stx2g*	-	-
ST281	Beef cattle	Harbin	2012	O22	H8	*stx1a*	*stx2d*	-	+
ST282	Cow	Heilongjiang	2012	O181	H4	-	*stx2d*	-	-
ST283	Cow	Heilongjiang	2012	ONT	H9	-	*stx2g*	-	-
ST284	Cow	Heilongjiang	2012	ONT	H9	-	*stx2g*	-	-
ST285	Cow	Heilongjiang	2012	O81	H31	-	*stx2d*	-	+
ST286	Cow	Heilongjiang	2012	O81	H31	-	*stx2d*	-	+
ST287	Beef cattle	Heilongjiang	2012	O99	H10	-	*stx2*	-	-
ST288	Cow	Heilongjiang	2012	O81	H31	-	*stx2d*	-	+
ST289	Marmota himalayana	Qinghai	2012	O112ab	H8	*stx1*	-	-	+
ST291	Marmota himalayana	Qinghai	2012	O5	HNT	*stx1*	-	-	+
ST292	Marmota himalayana	Qinghai	2012	O5	HNT	*stx1*	-	-	+
ST293	Marmota himalayana	Qinghai	2013	O53	H18	-	*stx2*	-	-
ST294	Marmota himalayana	Qinghai	2013	O53	H18	-	*stx2*	-	-
ST295	Marmota himalayana	Qinghai	2013	O53	H18	-	*stx2*	-	-
ST296	Marmota himalayana	Qinghai	2013	O71	H8	-	*stx2a*	-	+
ST298	Plateau Pika	Qinghai	2013	O74	H8	-	*stx2d*	-	+
ST299	Marmota himalayana	Qinghai	2013	O53	H18	-	*stx2*	-	-
ST300	Raw beef	Sichuan	2013	O96	H19	-	*stx2a*	-	+
ST301	pork	Sichuan	2013	O141	H29	-	*stx2e*	-	-

ST302	Raw beef	Sichuan	2013	O22	H16	-	*stx2b*	-	-
ST304	Raw beef	Beijing	2013	O40	H8	-	*stx2a*	-	+
ST305	Raw beef	Beijing	2013	O40	H8	-	*stx2a*	-	+
ST306	Food	Beijing	2013	O8	H9	-	*stx2e*	-	-
ST307	Food	Beijing	2013	O8	H9	-	*stx2e*	-	-
ST308	Food	Beijing	2013	O8	H9	-	*stx2e*	-	-
ST309	Diarrheal patient	Sichuan	2013	O48	H21	-	*stx2e*	-	-
ST310	Diarrheal patient	Shanghai	2013	O5	HNT	*stx1*	-	+	+
ST311	Diarrheal patient	Shanghai	2013	O68	H10	*stx1*	-	-	-
ST312	Diarrheal patient	Shanghai	2013	O26	H11	*stx1*	-	+	+
ST313	Raw beef	Beijing	2013	O100	H19	-	*stx2e*	-	-
ST314	Raw pork	Beijing	2013	O98	H10	-	*stx2e*	-	-
ST315	Raw pork	Beijing	2013	O98	H30	-	*stx2e*	-	-
ST316	Raw beef	Beijing	2014	O100	H19	-	*stx2e*	-	-
ST317	Raw mutton	Beijing	2014	ONT	H21	*stx1c*	-	-	-
ST318	Raw pork	Beijing	2014	O121	H10	-	*stx2e*	-	-
ST319	Raw mutton	Beijing	2014	O176	H4	*stx1c*	*stx2b*	-	+
ST320	Raw mutton	Beijing	2014	O5	H19	*stx1c*	*stx2b*	-	-
ST321	Raw mutton	Beijing	2014	O128	H2	*stx1c*	*stx2b*	-	+
ST322	Raw chicken meat	Beijing	2014	O176	H4	*stx1c*	-	-	+
ST323	Raw duck meat	Beijing	2014	O176	H4	*stx1c*	-	-	+
ST344	Raw mutton	Beijing	2014	O8	H30	-	*stx2e*	-	-
ST345	Raw pork	Beijing	2014	O57	H21	-	*stx2e*	-	-
ST346	Raw pork	Beijing	2014	O8	H19	-	*stx2e*	-	-
ST347	Raw beef	Beijing	2014	O110	H16	*stx1c*	-	-	-
ST348	Raw beef	Beijing	2014	O103	H25	*stx1a*	-	+	+
ST349	Raw beef	Beijing	2014	O98	H30	-	*stx2e*	-	-
ST350	Raw beef	Beijing	2014	O103	H25	*stx1a*	-	+	+
ST351	Raw beef	Beijing	2014	O98	H30	-	*stx2e*	-	-
ST352	Raw mutton	Beijing	2014	O120	H16	*stx1c*	-	-	-
ST353	Raw beef	Beijing	2014	O128	H2	*stx1c*	*stx2b*	-	-
ST354	Raw beef	Beijing	2014	O128	H2	*stx1c*	*stx2b*	-	-
ST355	Raw beef	Beijing	2014	O128	H2	*stx1c*	*stx2b*	-	-
ST356	Raw mutton	Beijing	2014	O161	H19	*stx1c*	-	-	+
ST357	Raw mutton	Beijing	2014	O176	H4	*stx1c*	-	-	+
ST358	Raw mutton	Beijing	2014	O104	H7	*stx1c*	-	-	-
ST359	Raw mutton	Beijing	2014	O128	H2	*stx1c*	*stx2b*	-	-
ST360	Raw mutton	Beijing	2014	O176	H4	*stx1c*	-	-	+
ST361	Raw mutton	Beijing	2014	O21	H25	*stx1a*	-	-	-
ST362	Raw mutton	Beijing	2014	O91	H14	*stx1a*	*stx2b*	-	-
ST363	Raw mutton	Beijing	2014	O104	H7	*stx1c*	-	-	-
ST364	Raw mutton	Beijing	2014	O128	H2	*stx1c*	*stx2b*	-	+
ST365	Raw beef	Beijing	2014	O12	HNT	-	*stx2c*	+	+

ST366	Raw beef	Beijing	2014	O128	H2	*stx1c*	-	-	-
ST367	Raw mutton	Beijing	2014	O100	H19	-	*stx2e*	-	-
ST368	Raw mutton	Beijing	2014	O104	H7	*stx1c*	-	-	-
ST369	Raw mutton	Beijing	2014	O22	H16	-	*stx2d*	-	+
ST370	Raw mutton	Sichuan	2014	O113	H7	*stx1c*	-	-	+
ST371	Raw beef	Sichuan	2014	O84	H20	*stx1d*	-	-	-
ST372	Raw beef	Sichuan	2014	O76	H21	*stx1a*	-	-	+
ST373	Raw mutton	Sichuan	2014	O21	H25	*stx1a*	-	-	-
ST374	Raw beef	Beijing	2014	O8	H30	-	*stx2a*	-	-
ST375	Raw beef	Beijing	2014	O20	H21	-	*stx2e*	-	-
ST376	Raw mutton	Beijing	2014	O128	H2	*stx1c*	*stx2b*	-	+
ST377	Raw mutton	Beijing	2014	O5	H9	*stx1a*	-	+	+
ST378	Raw beef	Beijing	2014	O116	H21	*stx1a*	*stx2a*	-	+
ST379	Raw pork	Beijing	2014	O8	H19	-	*stx2e*	-	-
ST380	Pig	Beijing	2014	O141	H29	-	*stx2e*	-	-
ST381	Raw mutton	Beijing	2014	O120	H30	-	*stx2e*	-	-
ST382	Pig	Beijing	2014	O91	H4	-	*stx2e*	-	-
ST383	Pig	Beijing	2014	O172	H30	-	*stx2e*	-	-
ST384	Raw pork	Beijing	2014	O8	H19	-	*stx2e*	-	-
ST385	Raw pork	Beijing	2014	O91	H4	-	*stx2e*	-	-
ST386	Raw pork	Beijing	2014	O91	H4	-	*stx2e*	-	-
ST387	Raw pork	Beijing	2014	O91	H4	-	*stx2e*	-	-
ST388	Pig	Guizhou	2012	O159	H16	-	*stx2e*	-	-
ST389	Pig	Guizhou	2012	O100	H20	-	*stx2e*	-	-
ST390	Pig	Guizhou	2012	ONT	H7	-	*stx2e*	-	-
ST391	Pig	Guizhou	2012	O87	H10	-	*stx2e*	-	-
ST392	Pig	Guizhou	2012	O100	H20	-	*stx2e*	-	+
ST393	Pig	Guizhou	2012	ONT	H7	-	*stx2e*	-	-
ST397	Diarrheal patient	Henan	2013	O130	H8	*stx1c*	-	-	-
ST398	Diarrheal patient	Henan	2013	O130	H8	*stx1c*	-	-	-
ST399	Diarrheal patient	Henan	2013	O130	H8	*stx1c*	-	-	-
ST400	Diarrheal patient	Henan	2013	O130	H8	*stx1c*	-	-	-
ST401	Diarrheal patient	Henan	2013	O130	H8	*stx1c*	-	-	-
ST402	Diarrheal patient	Henan	2013	O117	H8	*stx1c*	-	-	-
ST403	Diarrheal patient	Henan	2013	O117	H8	*stx1c*	-	-	-
ST404	Diarrheal patient	Henan	2013	O130	H8	*stx1c*	-	-	-
ST405	Diarrheal patient	Henan	2013	O117	H8	*stx1c*	-	-	-
ST406	Diarrheal patient	Shanghai	2012	O26	H11	*stx1a*	-	+	+
ST407	Diarrheal patient	Shanghai	2013	O5	HNT	*stx1a*	-	+	+
ST408	Diarrheal patient	Shanghai	2013	O21	H25	*stx1a*	-	-	-
ST409	Diarrheal patient	Shanghai	2013	ONT	H30	-	*stx2e*	-	-
ST410	Diarrheal patient	Shanghai	2013	O149	H10	*stx1c*	-	-	-
ST411	Diarrheal patient	Shanghai	2013	O26	H11	*stx1a*	-	+	+

ST412	Diarrheal patient	Shanghai	2013	O112ab	H8	*stx1a*	-	-	-
ST413	Diarrheal patient	Shanghai	2014	O20	H30	-	*stx2g*	-	-
ST414	Diarrheal patient	Shanghai	2014	O104	H7	*stx1c*	-	-	-
ST415	Diarrheal patient	Shanghai	2014	O84	H2	*stx1a*	-	+	+
ST416	Diarrheal patient	Shanghai	2014	O26	H11	*stx1a*	-	+	+
ST434	Healthy carrier	Guangdong	2014	O43	H2	-	*stx2d*	-	-
ST435	Healthy carrier	Qinghai	2013	O91	H14	*stx1a*	*stx2b*	-	+
ST437	Diarrheal patient	Guangdong	2010	O111	H8	*stx1a*	-	-	+
ST438	Healthy carrier	Guangdong	2014	ONT	H21	*stx1c*	-	-	-
ST439	Plateau Pika	Qinghai	2015	O169	H8	-	*stx2b*	-	+
ST440	Plateau Pika	Qinghai	2015	O74	H8	-	*2a+stx2d*	-	+
ST441	Plateau Pika	Qinghai	2015	O49	H21	-	*stx2b*	-	-
ST442	Plateau Pika	Qinghai	2015	O2	H45	*stx1a*	-	-	-
ST443	Plateau Pika	Qinghai	2015	O119	H19	*stx1a*	-	-	+
ST444	Plateau Pika	Qinghai	2015	O49	H21	-	*stx2b*	-	-
ST445	Plateau Pika	Qinghai	2015	O81	H21	-	*stx2a*	-	+
ST446	Plateau Pika	Qinghai	2015	O96	H8	-	*stx2d*	-	+
ST447	Plateau Pika	Qinghai	2015	O2	H45	*stx1a*	-	-	-
ST448	Plateau Pika	Qinghai	2015	O120	H9	*stx1a*	*stx2d*	-	+
ST449	Plateau Pika	Qinghai	2015	O163	19	-	*stx2b*	-	-
ST450	Plateau Pika	Qinghai	2015	O159	H21	*stx1a*	*stx2d*	-	+
ST451	Plateau Pika	Qinghai	2012	O8	H16	-	*stx2b*	-	-
ST452	Plateau Pika	Qinghai	2012	O8	H2	-	*stx2d*	-	+
ST453	Plateau Pika	Qinghai	2012	O8	H2	-	*stx2d*	-	+
ST454	Plateau Pika	Qinghai	2012	O8	H2	-	*stx2d*	-	+
ST455	Plateau Pika	Qinghai	2012	O82	H19	*stx1a*	-	-	+
ST456	Plateau Pika	Qinghai	2012	O2	H45	*stx1a*	-	-	-
ST457	Plateau Pika	Qinghai	2012	O170	H8	-	*stx2d*	-	+
ST458	Plateau Pika	Qinghai	2012	O74	H8	*stx1a*	*stx2d*	-	+
ST459	Plateau Pika	Qinghai	2012	O8	H2	-	*stx2d*	-	+
ST461	Beef cattle	Shandong	2015	O177	HNT	-	*stx2c*	+	+
ST463	Pig	Shandong	2015	O77	H30	-	*stx2e*	-	-
ST464	Pig	Shandong	2015	O77	H30	-	*stx2e*	-	-
ST465	Pig	Shandong	2015	O86	H32	-	*stx2e*	-	-
ST466	Pig	Shandong	2015	O44	H30	-	*stx2e*	-	-
ST467	Pig	Shandong	2015	O110 (O113)	H30	-	*stx2e*	-	-
ST468	Pig	Shandong	2015	O20 O60	H30	-	*stx2e*	-	-
ST469	Pig	Shandong	2015	O10	H30	-	*stx2e*	-	-
ST470	Pig	Shandong	2015	O110	HNT	-	*stx2e*	-	-
ST471	Pig	Shandong	2015	O114	H27	-	*stx2e*	-	-
ST472	Pig	Shandong	2015	O20	H27	-	*stx2e*	-	-
ST473	Pig	Shandong	2015	O65	H9	-	*stx2e*	-	-

ST474	Pig	Shandong	2015	O65	H9	-	*stx2e*	-	-
ST475	Pig	Shandong	2015	O8	HNT	-	*stx2e*	-	-
ST476	Tibetan antelope	Qinghai	2014	O102	H8	*stx1a*	-	-	-
ST477	Tibetan antelope	Qinghai	2014	O50	H14	*stx1a*	-	-	+
ST484	Tibetan antelope	Qinghai	2014	O105	H8	-	*stx2*	-	+
ST485	Tibetan antelope	Qinghai	2014	O74	H8	-	*stx2*	-	+
ST486	Tibetan antelope	Qinghai	2014	O74	H8	-	*stx2*	-	+
ST506	Diarrheal patient	Shandong	2014	O3	H4	*stx1*	-	-	-
ST507	Diarrheal patient	Shandong	2016	O3	H28	*stx1*	-	-	-
ST508	Diarrheal patient	Shanghai	2016	O26	H11	*stx1*	-	+	+
ST509	Healthy carrier	Guangdong	2016	O8	H19	-	*stx2*	-	-

Table S2. Characteristics of 138 *ehxA*-positive non-O157 STEC strains used in this study

Strain	Origin	Location	Year	O serogroup	H type	Hemolytic	Virulence genes						Multiplex-inc/rep PCR					*ehxA* genotype	*ehxA* group
							stx_1	stx_2	*eae*	*toxB*	*katP*	*espP*	I1-Ir	FIB	F	K	B/O		
STEC006	Goat	Henan	2009	O45	H2	Non	1c	2d	-	-	-	-	-	+	-	+	-	1	I
STEC007	Goat	Henan	2009	O91	H14	Narrow	1c	2d	-	-	-	+	-	+	+	-	-	2	I
STEC009	Goat	Henan	2009	O91	H14	Transparent	1c	2d	-	-	-	+	-	+	+	-	-	2	I
STEC011	Goat	Henan	2009	O103	H8	Non	1c	-	-	-	-	-	-	+	+	+	-	3	I
STEC015	Goat	Henan	2009	O103	H8	Non	1c	-	-	-	-	+	-	+	+	-	+	3	I
STEC016	Goat	Henan	2009	O103	H8	Non	1c	-	-	-	-	-	-	+	+	-	+	3	I
STEC017	Goat	Henan	2009	O141ac	H25	Non	-	2g	-	-	-	-	-	+	+	-	-	4	III
STEC023	Goat	Henan	2009	O103	H8	Narrow	1c	-	-	-	-	-	-	+	+	-	+	3	I
STEC024	Goat	Henan	2009	O103	H8	Transparent	1c	-	-	-	-	-	-	+	+	+	-	5	I
STEC025	Goat	Henan	2009	O103	H8	Transparent	1c	-	-	-	-	-	-	+	+	+	-	3	I
STEC026	Goat	Henan	2009	O103	H8	Narrow	1c	-	-	-	-	-	-	+	+	-	+	3	I
STEC027	Goat	Henan	2009	O103	H8	Narrow	1c	-	-	-	-	-	-	+	+	-	+	3	I
STEC033	Beef cattle	Heilongjiang	2009	O113	H19	Transparent	1a	2a	-	-	-	+	-	+	+	-	-	6	I
STEC036	Yak	Qinghai	2012	OONT	H21	Narrow	-	2d	-	-	-	+	-	+	+	-	-	7	I
STEC037	Yak	Qinghai	2012	OONT	H44	Transparent	1a	-	-	-	-	-	-	+	+	-	-	8	I
STEC038	Yak	Qinghai	2012	O8	H9	Narrow	1a	2d	-	-	-	+	-	+	+	-	-	7	I
STEC039	Yak	Qinghai	2012	OONT	H7	Transparent	1a	-	-	-	-	-	-	+	+	-	+	9	I
STEC040	Yak	Qinghai	2012	O6	H21	Non	1a	-	-	-	-	-	-	+	-	-	-	8	I
STEC042	Yak	Qinghai	2012	O78	H45	Transparent	-	2g	-	-	-	-	-	+	+	-	-	8	I

STEC043	Yak	Qinghai	2012	O8	H9	Narrow	1a	2d	-	-	-	+	-	-	+	-	-	7	I
STEC045	Yak	Qinghai	2012	O8	H9	Transparent	1a	2d	-	-	-	+	-	+	+	-	-	7	I
STEC046	Yak	Qinghai	2012	O6	NT	Non	1a	-	-	-	-	-	-	+	+	-	-	8	I
STEC047	Yak	Qinghai	2012	O165	H21	Non	1a	-	-	-	-	-	-	+	+	-	-	8	I
STEC049	Yak	Qinghai	2012	OONT	HNT	Narrow	-	2c	-	-	-	+	-	+	+	-	-	7	I
STEC050	Yak	Qinghai	2012	O8	H19	Narrow	1a	-	-	-	-	+	-	+	+	-	-	10	I
STEC051	Yak	Qinghai	2012	ONT	H21	Transparent	1a	-	-	-	-	-	-	+	+	-	-	3	I
STEC052	Yak	Qinghai	2012	ONT	H21	Transparent	1a	-	-	-	-	-	-	+	+	-	-	8	I
STEC053	Yak	Qinghai	2012	ONT	HNT	Narrow	1a	2a+2b	-	-	-	+	-	+	+	+	-	11	I
STEC054	Yak	Qinghai	2012	ONT	H8	Transparent	1a	2a+2b	-	-	-	+	-	+	+	+	-	12	I
STEC055	Yak	Qinghai	2012	O66	H8	Transparent	1a	-	-	-	-	+	-	-	+	-	-	13	I
STEC056	Yak	Qinghai	2012	O78	HNT	Narrow	-	2a	+	-	-	+	-	+	-	+	-	6	I
STEC057	Yak	Qinghai	2012	O78	H21	Transparent	-	2c	-	-	-	+	-	+	+	-	-	7	I
STEC063	Yak	Qinghai	2012	O8	H19	Transparent	1a	-	-	-	-	+	-	+	+	-	-	10	I
STEC064	Yak	Qinghai	2012	O8	H19	Transparent	1a	-	-	-	-	+	-	+	+	-	-	10	I
STEC065	Yak	Qinghai	2012	O8	H9	Narrow	1a	2d	-	-	-	+	-	+	+	-	-	7	I
STEC066	Yak	Qinghai	2012	O8	H9	Narrow	1a	2d	-	-	-	+	-	+	+	-	-	7	I
STEC068	Yak	Qinghai	2012	O78	H21	Narrow	-	2a	+	-	-	+	-	+	-	+	-	6	I
STEC071	Yak	Qinghai	2012	O8	H9	Transparent	1a	2d	-	-	-	+	-	+	+	-	-	7	I
STEC072	Yak	Qinghai	2012	O8	H9	Narrow	1a	2d	-	-	-	+	-	+	+	-	-	7	I
STEC073	Yak	Qinghai	2012	O8	H9	Narrow	1a	2d	-	-	-	+	-	+	+	-	-	7	I
STEC075	Yak	Qinghai	2012	O66	H21	Non	-	2a+2b	-	-	-	+	-	+	+	+	-	6	I
STEC076	Yak	Qinghai	2012	ONT	H8	Narrow	-	2b	-	-	-	-	-	+	+	-	-	14	I
STEC077	Yak	Qinghai	2012	O158	H8	Narrow	-	2b	-	-	-	-	-	+	+	-	-	14	I
STEC078	Yak	Qinghai	2012	O158	H8	Narrow	-	2b	-	-	-	-	-	+	+	-	-	15	I

STEC081	Yak	Qinghai	2012	O78	H8	Transparent	-	2a	-	-	-	-	-	-	-	-	-	16	I
STEC082	Yak	Qinghai	2012	O78	H8	Transparent	-	2a	-	-	-	-	-	+	-	-	-	16	I
STEC084	Yak	Qinghai	2012	O66	H21	Non	-	2a+2b	-	-	-	+	-	+	+	+	-	6	I
STEC089	Yak	Qinghai	2012	ONT	H21	Non	1a	-	-	-	-	-	-	+	+	-	-	17	I
STEC091	Yak	Qinghai	2012	ONT	H21	Transparent	1a	-	-	-	-	-	-	+	+	-	-	8	I
STEC092	Yak	Qinghai	2012	ONT	H8	Transparent	-	2b	-	-	-	-	-	+	+	-	-	14	I
STEC094	Yak	Qinghai	2012	O137	H21	Narrow	1a	2a	-	-	-	-	-	+	-	-	+	9	I
STEC095	Yak	Qinghai	2012	ONT	H8	Transparent	1a	-	-	-	-	-	-	+	+	-	-	16	I
STEC096	Yak	Qinghai	2012	O127	H8	Non	1a	2d	-	-	-	+	-	+	+	-	-	13	I
STEC100	Yak	Qinghai	2012	ONT	H8	Transparent	1a	2b	-	-	-	+	-	+	+	-	-	6	I
STEC101	Yak	Qinghai	2012	O8	H2	Transparent	-	2d	-	-	-	-	+	+	+	-	+	14	I
STEC102	Yak	Qinghai	2012	O165	H8	Transparent	1a	-	-	-	-	+	-	-	+	-	-	13	I
STEC103	Yak	Qinghai	2012	O165	H8	Non	1a	-	-	-	-	+	-	+	+	-	-	13	I
STEC115	Yak	Qinghai	2012	O6	H14	Transparent	-	2b	-	-	-	+	-	+	+	+	-	6	I
STEC116	Yak	Qinghai	2012	O6	H14	Transparent	-	2b	-	-	-	+	-	+	+	-	-	34	I
STEC119	Yak	Qinghai	2012	ONT	H8	Narrow	1a	2d	-	-	-	+	-	+	+	-	+	12	I
STEC120	Yak	Qinghai	2012	O165	H8	Transparent	1a	2d	-	-	-	+	-	+	-	+	-	12	I
STEC127	Yak	Qinghai	2012	O78	H8	Transparent	1a	2d	-	-	-	-	-	-	+	-	-	16	I
STEC128	Yak	Qinghai	2012	O78	H8	Narrow	1a	2d	-	-	-	-	-	+	-	-	-	16	I
STEC129	Yak	Qinghai	2012	ONT	H21	Non	1a	2d	-	-	-	-	-	+	-	-	+	9	I
STEC130	Yak	Qinghai	2012	ONT	H21	Non	1a	2d	-	-	-	-	-	+	+	-	-	9	I
STEC131	Yak	Qinghai	2012	ONT	H44	Transparent	1a	-	-	-	-	+	-	+	+	-	-	13	I
STEC132	Yak	Qinghai	2012	O123	H8	Transparent	1a	2b	-	-	-	+	-	+	+	-	-	6	I
STEC138	Yak	Qinghai	2012	O149	H45	Non	-	2d	-	-	-	-	-	+	+	-	-	16	I
STEC139	Yak	Qinghai	2012	O149	H45	Non	-	2d	-	-	-	-	-	-	+	-	-	16	I

STEC140	Yak	Qinghai	2012	ONT	H8	Narrow	-	2a	-	-	-	-	-	+	+	-	-	36	I
STEC141	Yak	Qinghai	2012	ONT	H8	Narrow	-	2a	-	-	-	-	-	+	+	-	-	14	I
STEC144	Yak	Qinghai	2012	O78	H45	Non	-	2a+2c	-	-	-	+	-	+	+	-	-	7	I
STEC145	Yak	Qinghai	2012	O78	H21	Non	-	2a+2c	-	-	-	+	-	+	+	-	-	7	I
STEC146	Yak	Qinghai	2012	O2	H21	Non	-	2a+2c	-	-	-	+	-	+	+	-	-	7	I
STEC152	Yak	Qinghai	2012	O78	H8	Transparent	-	2a	-	-	-	-	-	+	-	-	-	16	I
STEC153	Yak	Qinghai	2012	O78	H8	Narrow	-	2a	-	-	-	-	-	+	-	-	-	16	I
STEC155	Yak	Qinghai	2012	O12	H12	Non	1a	-	-	-	-	+	-	-	+	-	+	18	I
STEC156	Yak	Qinghai	2012	O78	H8	Transparent	-	2a	-	-	-	-	-	+	-	-	-	16	I
STEC158	Yak	Qinghai	2012	O78	H8	Transparent	-	2a+2c	-	-	-	+	-	+	+	+	-	19	I
STEC281	Beef cattle	Harbin	2012	O22	H8	Transparent	1a	2d	-	-	-	+	-	+	+	-	-	20	I
STEC285	Cow	Heilongjiang	2012	O81	H31	Narrow	-	2d	-	-	-	+	-	+	+	-	-	6	I
STEC286	Cow	Heilongjiang	2012	O81	H31	Transparent	-	2d	-	-	-	+	-	+	+	-	-	6	I
STEC288	Cow	Heilongjiang	2012	O81	H31	Transparent	-	2d	-	-	-	-	-	+	+	-	-	6	I
STEC289	Marmota himalayana	Qinghai	2012	O112ab	H8	Transparent	1	-	-	-	-	+	-	+	+	-	-	21	I
STEC291	Marmota himalayana	Qinghai	2012	O5	HNT	Transparent	1	-	-	-	-	+	-	+	+	-	-	22	II
STEC292	Marmota himalayana	Qinghai	2012	O5	HNT	Narrow	1	-	-	-	-	+	-	+	+	-	-	22	II
STEC296	Marmota himalayana	Qinghai	2013	O71	H8	Transparent	-	2a	-	-	-	+	-	+	+	-	-	8	I
STEC298	Plateau Pika	Qinghai	2013	O74	H8	Non	-	2d	-	-	-	+	-	+	+	+	-	23	I
STEC300	Raw beef	Sichuan	2013	O96	H19	Non	-	2a	-	-	-	-	-	-	-	-	-	24	I
STEC304	Raw beef	Beijing	2013	O40	H8	Non	-	2a	-	-	-	+	-	+	+	-	-	13	I
STEC305	Raw beef	Beijing	2013	O40	H8	Non	-	2a	-	-	-	+	-	+	+	-	+	13	I
STEC310	Diarrheal patient	Shanghai	2013	O5	HNT	Non	1	-	+	-	-	+	-	+	-	-	-	22	II
STEC312	Diarrheal patient	Shanghai	2013	O26	H11	Non	1	-	+	+	+	+	-	+	-	-	+	25	II
STEC319	Raw mutton	Beijing	2014	O176	H4	Transparent	1c	2b	-	-	-	-	-	+	+	-	-	3	I

STEC321	Raw mutton	Beijing	2014	O128	H2	Non	1c	2b	-	-	-	-	-	+	+	-	+	3	I
STEC322	Raw chicken meat	Beijing	2014	O176	H4	Narrow	1c	-	-	-	-	-	-	+	+	+	-	3	I
STEC323	Raw duck meat	Beijing	2014	O176	H4	Non	1c	-	-	-	-	-	-	+	+	-	-	3	I
STEC348	Raw beef	Beijing	2014	O103	H25	Narrow	1a	-	+	-	-	-	-	+	-	-	-	25	II
STEC350	Raw beef	Beijing	2014	O103	H25	Narrow	1a	-	+	-	-	-	-	+	-	-	-	26	II
STEC356	Raw mutton	Beijing	2014	O161	H19	Transparent	1c	-	-	-	-	-	-	-	-	-	-	35	I
STEC357	Raw mutton	Beijing	2014	O176	H4	Non	1c	-	-	-	-	-	-	+	-	-	-	3	I
STEC360	Raw mutton	Beijing	2014	O176	H4	Non	1c	-	-	-	-	-	-	-	+	-	-	27	I
STEC364	Raw mutton	Beijing	2014	O128	H2	Non	1c	2b	-	-	-	-	-	+	+	-	+	3	I
STEC365	Raw beef	Beijing	2014	O12	HNT	Transparent	-	2c	+	-	+	+	-	+	+	-	-	28	II
STEC369	Raw mutton	Beijing	2014	O22	H16	Transparent	-	2d	-	-	-	+	-	+	+	-	-	20	I
STEC370	Raw mutton	Sichuan	2014	O113	H7	Transparent	1c	-	-	-	-	-	-	+	+	-	-	29	I
STEC372	Raw beef	Sichuan	2014	O76	H21	Non	1a	-	-	-	-	+	-	-	-	+	-	30	I
STEC376	Raw mutton	Beijing	2014	O128	H2	Non	1c	2b	-	-	-	-	-	+	-	+	-	3	I
STEC377	Raw mutton	Beijing	2014	O5	H9	Non	1a	-	+	-	-	+	-	+	+	-	-	22	II
STEC378	Raw beef	Beijing	2014	O116	H21	Transparent	1a	2a	-	-	-	+	-	+	-	+	-	31	I
STEC392	Pig	Guizhou	2012	O100	H20	Non	-	2e	-	-	-	-	-	+	+	+	-	3	I
STEC406	Diarrheal patient	Shanghai	2012	O26	H11	Transparent	1a	-	+	+	+	+	-	+	-	-	+	25	II
STEC407	Diarrheal patient	Shanghai	2013	O5	HNT	Non	1a	-	+	-	-	+	-	+	-	-	-	22	II
STEC411	Diarrheal patient	Shanghai	2013	O26	H11	Non	1a	-	+	+	+	+	-	+	-	-	+	25	II
STEC415	Diarrheal patient	Shanghai	2014	O84	H2	Narrow	1a	-	+	-	-	-	-	+	+	-	-	32	II
STEC416	Diarrheal patient	Shanghai	2014	O26	H11	Transparent	1a	-	+	+	+	+	-	+	-	-	+	25	II
STEC435	Healthy carrier	Qinghai	2013	O91	H14	Narrow	1a	2b	-	-	-	-	-	+	+	-	-	3	I
STEC437	Diarrheal patient	Guangdong	2010	O111	H8	Transparent	1a	-	-	-	+	-	-	+	+	-	+	33	II
STEC439	Plateau Pika	Qinghai	2015	O169	H8	Transparent	-	2b	-	-	-	-	-	+	+	-	-	14	I

STEC440	Plateau Pika	Qinghai	2015	O74	H8	Narrow	-	2a+2d	-	-	-	+	-	+	+	+	-	23	I
STEC443	Plateau Pika	Qinghai	2015	O119	H19	Non	1a	-	-	-	-	+	-	-	+	-	+	10	I
STEC445	Plateau Pika	Qinghai	2015	O81	H21	Non	-	2a	-	-	-	+	-	+	-	+	-	12	I
STEC446	Plateau Pika	Qinghai	2015	O96	H8	Transparent	-	2d	-	-	-	-	-	+	+	-	-	14	I
STEC448	Plateau Pika	Qinghai	2015	O120	H9	Transparent	1a	2d	-	-	-	+	-	+	+	-	-	7	I
STEC450	Plateau Pika	Qinghai	2015	O159	H21	Transparent	1a	2d	-	-	-	+	-	-	-	-	-	9	I
STEC452	Plateau Pika	Qinghai	2012	O8	H2	Narrow	-	2d	-	-	-	-	-	+	+	-	-	14	I
STEC453	Plateau Pika	Qinghai	2012	O8	H2	Narrow	-	2d	-	-	-	-	-	+	+	-	-	14	I
STEC454	Plateau Pika	Qinghai	2012	O8	H2	Narrow	-	2d	-	-	-	-	-	+	+	-	-	14	I
STEC455	Plateau Pika	Qinghai	2012	O82	H19	Transparent	1a	-	-	-	-	+	-	+	+	-	+	10	I
STEC457	Plateau Pika	Qinghai	2012	O170	H8	Transparent	-	2d	-	-	-	-	-	+	+	-	-	14	I
STEC458	Plateau Pika	Qinghai	2012	O74	H8	Narrow	1a	2d	-	-	-	-	-	+	-	-	-	16	I
STEC459	Plateau Pika	Qinghai	2012	O8	H2	Non	-	2d	-	-	-	-	-	+	+	-	-	14	I
STEC461	Beef cattle	Shandong	2015	O177	HNT	Non	-	2c	+	-	+	+	-	+	+	-	-	28	II
STEC477	Tibetan antelope	Qinghai	2014	O50	H14	Non	1a	-	-	-	-	+	-	+	+	-	-	2	I
STEC484	Tibetan antelope	Qinghai	2014	O105	H8	Non	-	2	-	-	-	-	-	+	+	+	-	14	I
STEC485	Tibetan antelope	Qinghai	2014	O74	H8	Transparent	-	2	-	-	-	+	-	-	+	+	-	23	I
STEC486	Tibetan antelope	Qinghai	2014	O74	H8	Narrow	-	2	-	-	-	+	-	-	+	+	-	23	I
STEC508	Diarrheal patient	Shanghai	2016	O26	H11	Non	1	-	+	+	+	+	-	+	-	-	-	25	II
